# Contributions of Histone H3 Nucleosome Core Surface Mutations to Chromatin Structures, Silencing and DNA Repair

**DOI:** 10.1371/journal.pone.0026210

**Published:** 2011-10-28

**Authors:** Michel Fink, Jeffrey S. Thompson, Fritz Thoma

**Affiliations:** 1 Department of Biology, Institute of Cell Biology, ETH Zurich, Zurich, Switzerland; 2 Department of Biology, Denison University, Granville, Ohio, United States of America; George Mason University, United States of America

## Abstract

Histone H3 mutations in residues that cluster in a discrete region on the nucleosome surface around lysine 79 of H3 affect H3-K79 methylation, impair transcriptional silencing in subtelomeric chromatin, and reveal distinct contributions of histone H3 to various DNA-damage response and repair pathways. These residues might act by recruitment of silencing and DNA-damage response factors. Alternatively, their location on the nucleosome surface suggests a possible involvement in nucleosome positioning, stability and nucleosome interactions. Here, we show that the yeast H3 mutants *hht2*-T80A, *hht2*-K79E, *hht2*-L70S, and *hht2*-E73D show normal nucleosome positioning and stability in minichromosomes. However, loss of silencing in a subtelomeric *URA3* gene correlates with a shift of the promoter nucleosome, while nucleosome positions and stability in the coding region are maintained. Moreover, the H3 mutants show normal repair of UV lesions by photolyase and nucleotide excision repair in minichromosomes and slightly enhanced repair in the subtelomeric region. Thus, these results support a role of those residues in the recruitment of silencing proteins and argue against a general role in nucleosome organization.

## Introduction

Chromatin structure serves as a central regulator for DNA-associated cellular processes in eukaryotic cells, including transcription, replication, and repair. The primary components of chromatin are the highly conserved histone proteins, which provide the structural foundation for the organization of DNA. Molecular genetic, biochemical and genome wide studies have elucidated many aspects of the structural and functional roles of histones in genome organization. Here we investigate a set of nucleosome surface mutants in yeast with respect to their potential impact on chromatin structure, silencing and DNA repair.

Eukaryotic genomes are folded in arrays of nucleosome cores connected by linker DNA and further condensed into compact fibers and additional levels of higher order structures. While nucleosome cores are formed by intranucleosomal histone-histone and histone-DNA contacts, the close proximity of nucleosomes in arrays and higher order structures may require internucleosomal interactions of histones and DNA as well as a contribution of non-histone chromosomal proteins [1], [2]. Nucleosome cores are cylindrical particles containing of about 147 bp DNA wrapped around an octamer of core histones, two each of H2A, H2B, H3, and H4. The histones are folded in the centre of the core and have flexible N-terminal tails that protrude from the particle [3]. The tails may interact with the DNA or histones of adjacent nucleosomes, thereby contributing to higher order structures [4], [5]. The histone fold domains show an irregular surface with a distinct charge distribution that has also been implicated in nucleosome-nucleosome contacts to promote chromatin higher order structure formation [3], [4], [6].

In chromatin of the budding yeast *S. cerevisiae*, nucleosome cores are connected by short linker DNA [7], [8] and reduced cleavage by micrococcal nuclease (MNase) between nucleosomes suggested that some nucleosomes can be in close face-to-face contact [9], [10], [11], [12]. The nucleosome arrays are frequently interrupted by nucleosome free regions (NFRs) that occur at promoters and 3′ ends of genes and at origins of replication. A major fraction of nucleosomes is positioned [8], [13], [14]. Positioning is mediated by boundaries such as NFRs that restrict statistical distribution of nucleosomes, the DNA-sequence, and chomatin folding [8], [9], [11], [12], [13], [14], [15], [16], [17], [18], [19], [20]. Despite the detailed information on the genome wide arrangement of nucleosomes in yeast, the folding into higher order structures remains unclear and evidence supporting compact fibers in yeast is controversial [21], [22], [23].

Heterochromatin is a specialized higher order chromatin structure that restricts access of proteins to DNA and silences gene expression. In *S. cerevisiae* silent chromatin regions are found at telomeres, at the silent mating-type loci, and at the ribosomal DNA. Subtelomeric silencing depends on spreading of the silencing complex, which consists of Sir2, Sir3, and Sir4 proteins, from the telomeres and requires the histone deacetylase activity of Sir2 [24]. Genetic screens have identified H3 and H4 residues on a specific surface located at the H3-H4 histone-fold motif, which are important for transcriptional silencing of RNA polymerase II dependent reporter genes. Mutated amino acid residues that impaired silencing cluster around and include lysine 79 of H3 (H3-K79) [25], [26], [27]. H3-K79 is a site for methylation, by the conserved histone methyltransferase Dot1. Dot1 can add 1–3 methyl groups per residue, influenced in part by another histone modification, ubiquitylation of H2B [28]. H3-K79 is hypermethylated in transcriptionally active and hypomethylated in silenced chromatin, suggesting that hypermethylation of histone H3-K79 limits silencing to discrete loci by preventing the binding of Sir proteins elsewhere along the genome [29], [30]. Recent work established that Sir3 binds two locations on the nucleosome core, the LRS surface (Loss of Ribosomal DNA Silencing) and the N-terminal histone tails [31], [32], [33], [34].

In addition to silencing, some of the histone mutants compromise the DNA damage response. UV irradiation causes the formation of cyclobutane pyrimidine dimers (CPDs) and pyrimidine- (6-4)-pyrimidone photoproducts (6-4 PPs), which both can be repaired by nucleotide excision repair (NER). In yeast, CPDs can also be repaired by a CPD specific photolyase that reverses the damage in a light-dependent reaction (photoreactivation, PR). Unrepaired lesions can be tolerated and bypassed by post replication repair (PRR) pathways [35], [36]. Deletion of *DOT1* results modest sensitivity towards UV and ionizing irradiation, and increased resistance to methylmethane sulfonate (MMS), suggesting that methylation of H3-K79 plays distinct roles in the repair of specific forms of DNA damage [37], [38], [39], [40]. Histone H3 point mutations identified in the vicinity of H3-K79 that have distinct effects on H3-K79 methylation states showed varying degrees of UV-sensitivity and genetic interactions with UV-damage response pathways, suggesting that H3-K79 methylation states may be modulated in response to UV damage via a trans-histone regulatory pathway. In particular, *hht2*-L70S and *hht2*-T80A were found to cause no additional UV-sensitivity when combined with an NER mutation (*rad1*) and therefore act within the NER pathway, while *hht2*-E73D revealed additional UV-sensitivity indicating a role outside of NER [41].

While such genetic analysis has contributed to the identification of functionally important histone domains, many questions remain as to the mechanism by which these domains operate. They may serve as binding sites for proteins involved in silencing and the DNA damage response. On the other hand, residues that map on the histone octamer surface might play a role in establishing contacts between nucleosomes in arrays and higher order structures, thereby affecting nucleosome positioning and stability. We therefore investigated whether mutations that affect silencing and repair (*hht2*-T80A, *hht2*-K79E, *hht2*-L70S, *hht2*-E73D) do so as a consequence of altered nucleosome arrangements. We show that all mutants maintained nucleosome positions and stability as well as the capacity to repair UV-lesions. However, loss of silencing in the subtelomeric *URA3* gene correlated with an altered position of the promoter nucleosome and slightly enhanced repair of subtelomeric chromatin. These results support a role of those residues in the recruitment of silencing proteins and argue against a general role in nucleosome organization.

## Results

### Histone H3 mutants maintain nucleosome positioning and stability in minichromosomes

**Figure 1:**
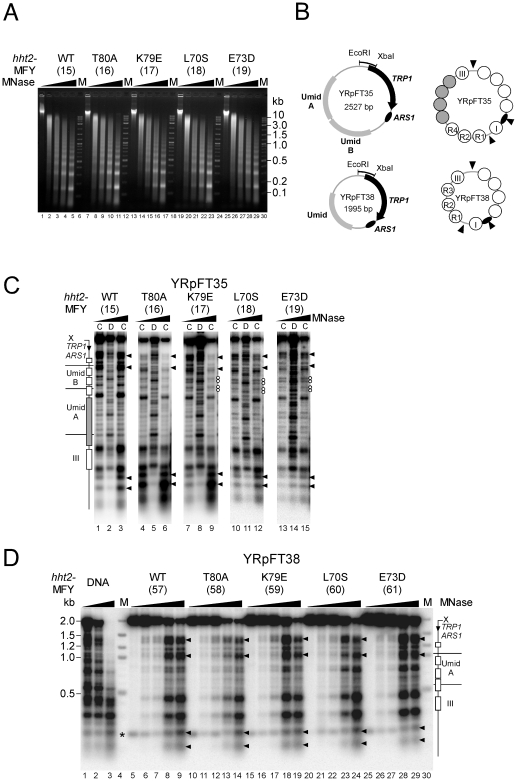
Chromatin structures preserved in *hht2* mutants. (**A**) Nucleosome repeats of genomic chromatin. Nuclei from *HHT2* (MFY15), *hht2*-T80A (MFY16), *hht2*-K79E (MFY17), *hht2*-L70S (MFY18), and *hht2*-E73D (MFY19) were digested with MNase (0, 25, 50, 100 and 200 U/ml). DNA was purified and separated on a 1% agarose gel containing ethidium bromide. M is a 2 log DNA ladder (New England BioLabs). (**B**) Schematic representation of the minichromosomes YRpFT35 and YRpFT38. The minichromosomes contain one (Umid) and two copies (UmidA and UmidB) of sequences of the *URA3* coding region (Umid) inserted in the TRP1ARS1 circle. Indicated are: the *TRP1* gene, the autonomously replicating sequence *ARS1*, the EcoRI-XbaI fragment used to generate probes for indirect end-labeling; nucleosome positions (circles) determined by MNase digestion. Four nucleosomes are tightly packed in YRpFT35 forming a tetranucleosome (dark circles) [10], [42]. (**C**) Nucleosome footprints in YRpFT35. Chromatin (C lanes) and DNA (D lanes) was digested with MNase, cut with XbaI, run on a 1% agarose gel, blotted and hybridized with the XbaI-EcoRI probe. Wedges on top of the lanes denote increasing MNase concentrations; rectangles mark the positions of nucleosomes; the dark rectangle indicates the footprint of the tightly packed tetranucleosome; an arrow denotes the direction of *TRP1* transcription; X indicates the XbaI site. Arrowheads indicate open, non-nucleosomal regions; white dots point to double bands possibly originating from alternative nucleosome positions. (**D**) Nucleosome footprints in YRpFT38. Chromatin and DNA was analyzed as in (C). M is a 2 log DNA ladder. The asterisk denotes a cross-hybridization with genomic DNA.

To investigate whether histone H3 mutations in close proximity to methylatable H3-K79 (*hht2*-T80A, *hht2*-K79E, *hht2*-L70S, and *hht2*-E73D; hereafter abbreviated *hht2* mutants) affect chromatin structure, we used yeast strains in which both genomic loci coding for histones H3 and H4 (*HHT1-HHF1* and *HHT2-HHF2*) were disrupted and replaced with either an *HHT2* wild-type or an *hht2* mutant allele of H3 on a centromeric plasmid [26]. The relative expression levels of wildtype H3 and the H3 mutants were very similar [41]. To test whether these H3 mutations affect the stability of nucleosomes, nucleosome positioning, and nucleosome-nucleosome contacts, the strains were transformed with circular minichromosomes (YRpFT35 and YRpFT38; Figure 1 B) that were shown to have distinct chromatin structures in strains containing the wild type set of histone genes (S288c) [10]. Both minichromosomes were generated by insertion of one (YRpFT38) or two tandem copies (YRpFT35) of Umid sequences (corresponding to 3.5 nucleosomes of the *URA3* coding region) in the TRP1ARS1 circle. Both minichromosomes showed (i) nuclease sensitive regions (NSRs) at the *TRP1* promoter (EcoRI site), at the origin of replication (*ARS1*), and a non-functional NSR at one end of Umid; (ii) four imprecisely positioned nucleosomes on *TRP1*; and (iii) positioned nucleosomes (I and III, R1, R2, R3, R4) in an untranscribed region. In addition, YRpFT35 revealed a long nuclease-resistant footprint in UmidA and the flanking region consistent with four close-packed nucleosomes forming a “tetranucleosome”. Different structures on the two Umid sequences in YRpFT35 demonstrate that the DNA sequence does not determine nucleosome positioning in these regions. The presence of positioned and tightly packed nucleosomes and nuclease-sensitive regions makes these minichromosomes suitable substrates to test nucleosome positioning, stability, and nucleosome-nucleosome contacts. Moreover, the extrachromosomal nature of those circular minichromosomes allows one to investigate structures independent of chromosomal position effects and to use supercoiling assays in addition to conventional nuclease digestions [42].

Chromatin was digested with increasing amounts of MNase; DNA was purified, separated on a 1% agarose gel, and stained with ethidium bromide. Bulk chromatin from all tested stains displayed clear nucleosomal ladders and a similar accessibility to MNase (Figure 1 A) indicating that the H3 mutations do not affect global chromatin organization.

To investigate the arrangement of the nucleosomes in the minichromosomes, MNase cutting sites were displayed by indirect end labeling from the XbaI site of YRpFT35 and YRpFT38 (Figure 1 C and D). Cleavage sites in chromatin were compared with those in naked DNA. Regions that were protected from cleavage in chromatin and encompass 140–160 bp were interpreted as positioned nucleosomes [43]. The cutting patterns in YRpFT35 and YRpFT38 were similar in all tested strains and therefore independent of the histone mutation (Figure 1 C and D). Nucleosome footprints (boxes in Figure 1 C and D) were readily identified as well as a long footprint characteristic for the “tetranucleosome” in YRpFT35 (dark box in UmidA in Figure 1 C) and the nuclease sensitive regions (arrow heads). Only very subtle differences were noticed in UmidB nucleosomes of YRpFT35 between the strains expressing the various *hht2* alleles. The *hht2*-K79E (MFY17), *hht2*-L70S (MFY18), and *hht2*-E73D (MFY19) strains showed two double bands of similar intensities (Figure 1 D, white dots), while in the *HHT2* (MFY15) and the *hht2*-T80A (MFY16) strain the upper bands of the double bands were more pronounced. Thus, the *hht2* mutants did not dramatically affect positions of spaced nucleosomes, nor contacts of tightly packed nucleosomes, nor the nuclease sensitive regions (*ARS1* and *TRP1* promoter). Moreover, the strong footprints indicate that nucleosomes were not remarkably destabilized.

**Figure 2:**
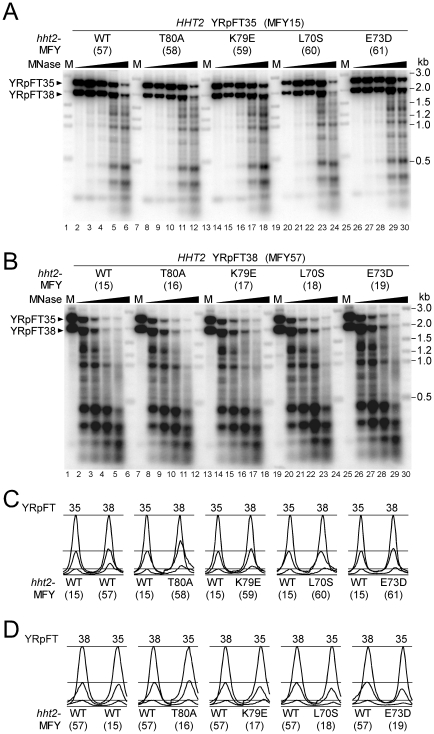
Co-digestion analyses of chromatin from *hht2* mutants. (**A**) Equal amounts of nuclei from *HHT2* cells containing YRpFT35 (MFY15) and nuclei from either *HHT2* (MFY57), *hht2*-T80A (MFY58), *hht2*-K79E (MFY59), *hht2*-L70S (MFY60), or *hht2*-E73D (MFY61) cells containing YRpFT38 were mixed and codigested with MNase (0, 5, 10, 25, and 50 U/ml). Nucleosome footprints were detected as in Figure 1. (**B**) Equal amounts of nuclei from *HHT2* cells containing YRpFT38 (MFY57) and nuclei from either *HHT2* (MFY15), *hht2*-T80A (MFY16), *hht2*-K79E (MFY17), *hht2*-L70S (MFY18), or *hht2*-E73D (MFY19) cells containing YRpFT35 were mixed and codigested with MNase (0, 25, 50, 100, and 200 U/ml). (**C**) and (**D**) Phosphorimager scans of the top two bands representing the linearized YRpFT35 and YRpFT68 from the samples digested with 0, 25, and 50 U/ml MNase of the blots shown in panels A and B, respectively. The values were normalized with respect to the band intensities of the undigested samples.

Since MNase hydrolyses DNA and RNA [44] and since different yeast strains and chromatin preparations might vary in RNA contents, we performed codigestion experiments with MNase [42] to quantitatively compare the stability of chromatin containing mutant H3 histones with wild-type chromatin. Nuclei from *HHT2* wild-type cells containing YRpFT35 (MFY15) were mixed and codigested with nuclei from cells expressing a wild type or one of the *hht2* alleles and containing YRpFT38. Digestion kinetics was assessed by indirect end labeling with a *TRP1* fragment that detects both minichromosomes (Figure 2 A). *Vice versa*, the experiment was repeated with wild type cells containing the short minichromosome YRpFT38 (MFY57) and mutants containing the long minichromosome YRpFT38 (Figure 2 B). In both co-digestion series, the top bands representing the undigested YRpFT35 and YRpFT38 minichromosomes decreased with similar kinetics. Scans of the top bands of the samples digested with 0, 25, and 50 U/ml MNase did not manifest pronounced differences in chromatin susceptibility to degradation by MNase (Figure 2 C and D). Subtle differences observed in T80A (Figure 2C), L70S, E73D, K79E (Figure 2 D) were not verified in the complementary codigestion. Taken together, the *hht2* mutations do not obviously change the accessibility of chromatin to MNase nor affect characteristic features of chromatin in minichromosomes.

**Figure 3:**
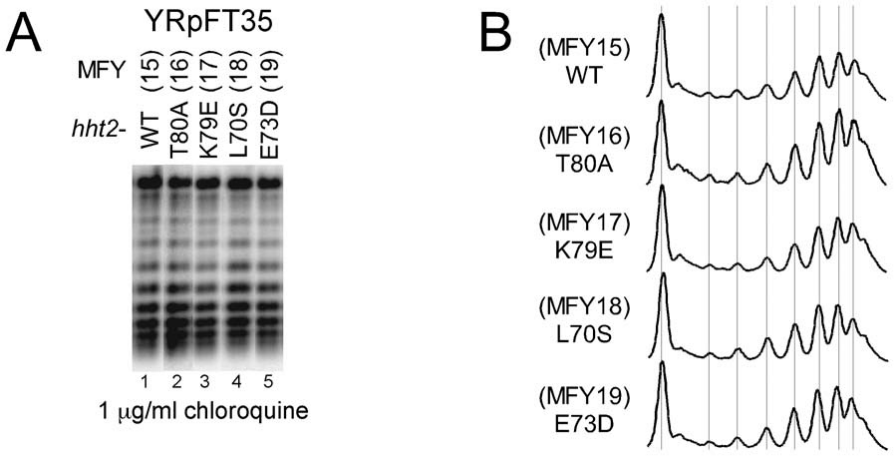
Superhelical densities of the minichromosome YRpFT35 isolated from *hht2* mutant strains. (**A**) DNA of the indicated strains was electrophoresed on a 0.75% agarose gel containing 1 µg/ml chloroquine and transferred to a Zeta-Probe GT membrane. YRpFT35 superhelical density was detected by hybridization with the EcoRI-XbaI probe of *TRP1* (see Figure 1). (**B**) Scans of lanes 1 to 5 (A).

As an alternative assay to investigate chromatin and nucleosomes stability *in vivo*, we tested the superhelical density of the circular minichromosome YRpFT35. To this end, DNA was purified from wild type and *hht2* mutant cells. Plasmid topoisomers were separated in chloroquine-agarose gels and analyzed by Southern blotting. As shown in Figure 3, the topoisomer distributions of YRpFT35 were similar in all mutants and indistinguishable from the wild type strain. The results confirm that none of the *hht2* mutations substantially destabilized nucleosomes nor affected nucleosome density in YRpFT35.

### The H3 mutations maintain nucleosome positioning in the coding region of a subtelomeric *URA3* gene but alter the position of the promoter nucleosome

Genes placed near telomeres in *S. cerevisiae* are transcriptionally repressed by binding of a silencing complex containing Sir2, Sir3 and Sir4 proteins [45], [46], [47]. The *hht2* mutants used in this study were identified in a screen for loss of silencing of a *URA3* reporter gene that was inserted at the telomere on the left arm of chromosome VII (TEL-VII-L) [26]. The results are consistent with the observation that the surface on the nucleosome around K79 serves as a binding site for Sir3 [48], [49] and that loss of subtelomeric *URA3* silencing in the *hht2* mutants is due to an inability of the silencing complex to bind and spread to this region [50]. Alternatively, it is also conceivable that the *hht2* mutations affect chromatin structure in subtelomeric regions and/or that loss of silencing proteins affects chromatin structure. In support of this idea, a recent chromatin excision approach revealed differential contributions of H3 and H4 residues on conformational properties of heterochromatin [51].

**Figure 4:**
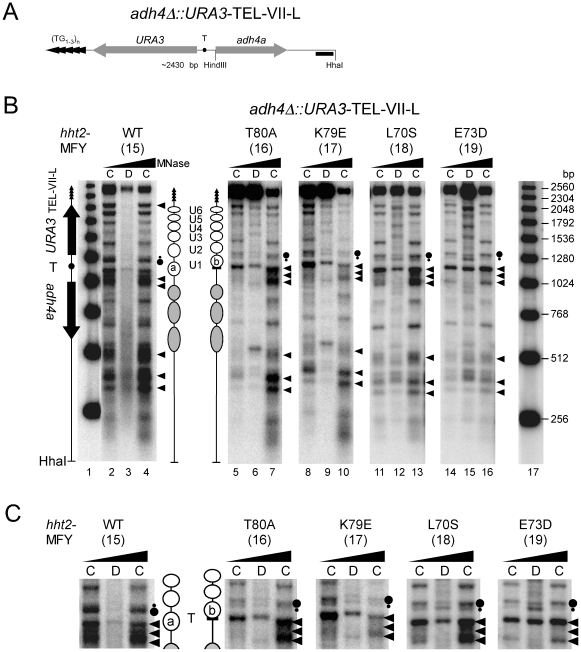
H3 dependent chromatin changes in a subtelomeric *URA3* gene. (**A**) Schematic representation of the left end of chromosome VII. The *URA3* gene was integrated at the HindIII site of *ADH4* locus [69]. Shown are the HhaI restriction fragment including the 3′-end of *ADH4* (*adh4a*), *URA3* with the promoter containing the TATA box (T), telomere repeats (TG_1–3_)_n_ (arrow heads), the end-label probe (black bar). (**B**) Nucleosome footprints. Chromatin (C lanes) and DNA (D lanes) were digested with MNase (as in Figure 1). DNA was purified, digested with HhaI, separated on a 1% agarose gel, blotted and hybridized with a probe close to the HhaI site. Indicated are positioned nucleosomes (circles). Big and small dots at the 5′-end of *URA3* next to the lanes indicate efficient and reduced cutting, respectively. (**C**) Enlarged section of the *URA3* promoter and 5′ coding region.

We therefore investigated chromatin footprints of *URA3* at the telomere TEL-VII-L (Figure 4). The results revealed positioned nucleosomes in the *adh4a* region and characteristic chromatin structures of the *URA3* gene as observed previously in the normal locus in chromosome V, the subtelomeric regions on chromosomes V, and in various minichromosomes, namely a nuclease sensitive promoter (arrow heads) and six positioned nucleosomes (U1 to U6) and a nuclease sensitive 3′end (arrow head). Nucleosome U1 is flanked by the TATA box and contains the major transcription start sites [9], [12], [19], [20]. The arrangement of the nucleosomes was similar in all tested strains. Therefore the *hht2* mutants do not disrupt chromatin structure near the telomere, which complements the results obtained with the minichromosome.

There was however a significant difference in positioning of the promoter nucleosome U1 between wild type and mutant cells. Nucleosome U1 at the 5′ end can occupy two extreme positions (circles a and b), defined by double bands at the 5′ end of the gene [12], [19]. In *HHT2* wild-type cells (MFY15) the nucleosome (U1a) partially protects the cutting site at the TATA box and the lower band of the double bands at the 5′ end is more intense (Figure 4 C). On the other hand, all *hht2* mutants (MFY16, MFY17, MFY18, and MFY19) showed a stronger cut at the TATA box relative to other sites and a more pronounced upper band of the double bands at the 5′ end. This indicates that in cells where *URA3* was silenced (*HHT2*) the nucleosome preferentially adopts position U1a covering the TATA box, whereas in the *hht2* mutants, when *URA3* is transcribed, this nucleosome was shifted towards the coding region to position U1b exposing the TATA-box. A similar nucleosome shift at the 5′ end of a subtelomeric *URA3* gene was previously found in *sir3Δ* cells [19] suggesting that the rearrangement of the nucleosome at the *URA3* promoter depends on binding of silencing proteins to this region. Whether this change of U1 is a cause or consequence of transcription is unknown. However, the fact that only one nucleosome was affected argues against a general role of the H3 mutants in nucleosome stability and nucleosome contacts.

### Telomere length is not affected by the H3 mutations

Telomeres are nucleoprotein structures at the end of chromosomes that protect the chromosome ends from degradation, end-to-end-fusion, and play additional roles in genome stability and subtelomeric transcriptional silencing. In *S. cerevisiae*, telomeric DNA is composed of arrays of heterogeneous TG_1–3_ sequences, normally about 300 bp in length. The length of the telomeric DNA repeats, which are generated by the activity of telomerase, is regulated by the action of various factors including histone-modifying enzymes. Telomeres serve as nucleation sites for subtelomeric heterochromatin formation, and elongation of telomeric DNA increases subtelomeric silencing, whereas several mutations that disrupt telomeric silencing also decrease the length of telomeres [52], [53], [54], [55], [56].

**Figure 5:**
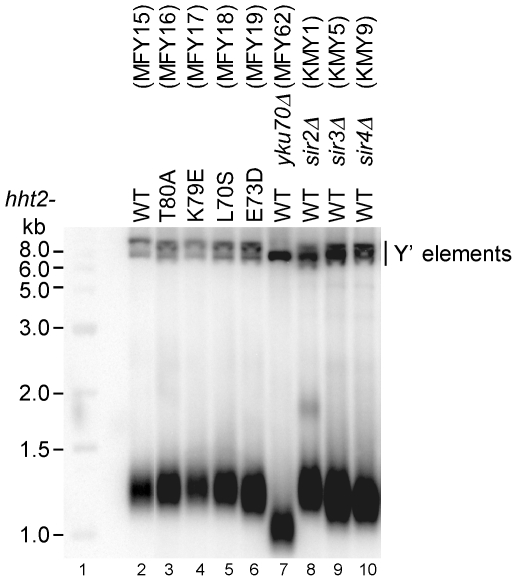
Analysis of telomere length in *hht2* mutants. The DNA from the indicated strains was isolated, digested with XhoI, separated on a 1% agarose gel and transferred to a Zeta-Probe GT membrane and hybridized with a Y′-specific probe (lanes 2–10). Lane 1 shows a molecular size marker.

To investigate whether loss of subtelomeric silencing in *hht2* mutants correlates with changes in length of telomeric DNA, we grew yeast cultures in YPD to an A_600_ of about 2 and isolated DNA. The DNA was then digested with XhoI, separated on a 1% agarose gel and the length of telomeric DNA was analyzed by Southern blotting with a Y′-specific probe (Figure 5). Strains with *sir3Δ*, *sir4Δ* and *yku70Δ* mutations revealed telomere shortening as described [55], [56], but the *hht2* mutants as well as *sir2Δ* maintained the normal telomere length. Thus, loss of subtelomeric silencing in the *hht2* mutants is not accompanied by significant changes in length of telomeric DNA.

### The *hht2* mutants show normal accessibility of UV lesions to photolyase and nucleotide excision repair (NER)

Among the mutants used here, *hht2*-L70S, *hht2*-E73D, *hht2*-T80A showed varying degrees of UV-sensitivity and genetic interactions with UV-damage response pathways. In particular, *hht2*-L70S and *hht2*-T80A were found to act within the NER pathway, while *hht2*-E73D revealed increased UV-sensitivity, indicating an additional role outside of NER [41]. Since packaging of DNA in chromatin affects accessibility and repair of UV lesions [57], we analyzed repair of CPDs by NER and photolyase to assess the impact the H3 mutants.

**Figure 6:**
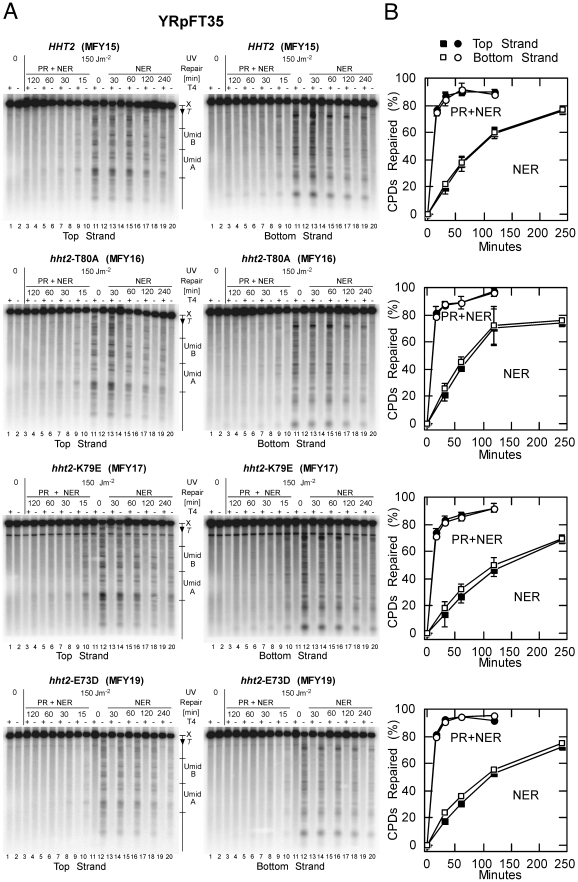
Efficient repair of CPDs by photolyase and NER in YRpFT35. Yeast cells containing YRpFT35 were irradiated with 150 J/m^2^ of UV light, either put on ice to measure the initial damage (lanes 11 and 12), exposed to photoreactivating light (PR+NER) for 15 to 120 minutes (lanes 3 to 10), or kept in the dark for NER (30 to 240 minutes, lanes 13 to 20). The DNA was purified, cut at CPDs with T4-endonuclease V (T4+, odd lanes) or mock treated without T4-endonuclease V (T4−, even lanes). To display the CPD cutting sites in YRpFT35, the DNA was digested with XbaI (X), separated on 1.5% alkaline agarose gels, transferred to Zeta-Probe GT membranes and hybridized with strand specific EcoRI-XbaI probes (Figure 1). (**A**) Phosphorimages of the top and bottom strand. (**B**) Quantitative analysis of CPD repair in YRpFT35. Shown are averages of four gels (MFY15–17) and two gels (MFY19) of one UV experiment each.

**Figure 7:**
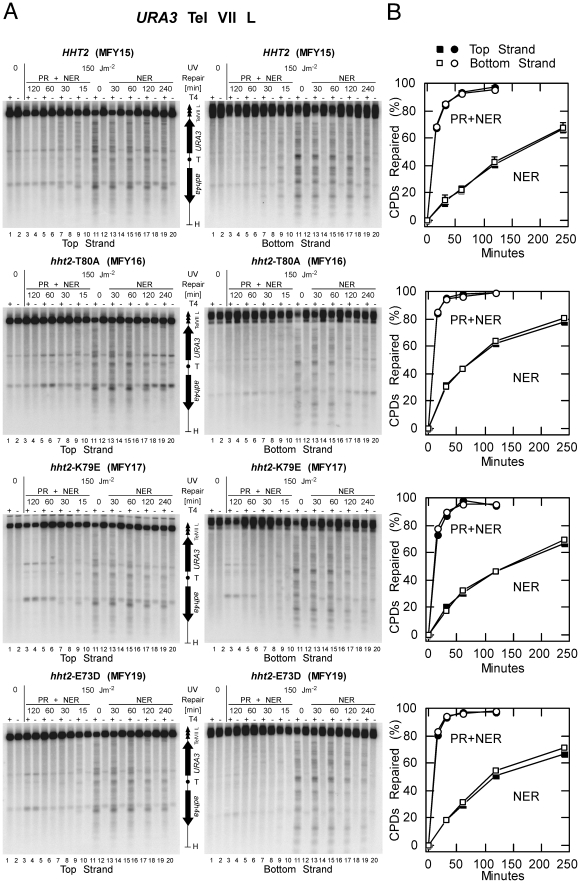
Repair of CPDs in subtelomeric chromatin. DNA repair experiments were done as described in Figure 6. To analyze CPD repair on the *adh4Δ::URA3-TEL*-VII-L locus the DNA was digested with HhaI (H) separated on 1.5% alkaline agarose gels, transferred to Zeta-Probe GT membranes and hybridized with strand specific probes close to the HhaI site (Figure 4). (**A**) Phosphorimages of top and bottom strand. Asterisks indicate cross-hybridization to unknown restriction fragments. (**B**) Quantitative analysis of CPD repair of *adh4Δ::URA3*-TEL-VII-L. Shown are averages of four gels (MFY15) and two gels (MFY16, MFY17, MFY19) of one UV experiment each.

To measure DNA repair of UV lesions, yeast cultures were UV-irradiated with 150 J/m^2^ to generate about 0.3 CPDs/kb, and either exposed to light for photoreactivation or incubated in the dark for NER. DNA was purified, cut at CPDs with T4-endonuclease V and the cutting sites were displayed by indirect endlabelling using alkaline gels and strand specific probes for YRpFT35 and the subtelomeric *URA3* region (Figures 6 and 7).

DNA of non-irradiated cells showed the intact restriction fragment (top band) irrespective of T4-endonuclease V treatment (Figures 6 A and 7 A, lanes 1 and 2). DNA of irradiated cells showed the fraction of intact restriction fragments in the mock-treated lanes (−T4 EndoV) and a specific band pattern and a weaker top band when cut at CPDs with T4-endonuclease V (+T4 EndoV). The bands represent the yields and distribution of CPDs along the DNA fragment. Top and bottom strands revealed different patterns demonstrating strand specificity. CPD bands disappeared rapidly when cells were exposed to photoreactivating light (Figures 6 A and 7 A, lanes 3 to 9) due to the combined activity of photolyase and NER (PR+NER). The bands disapeared more slowly by NER alone when cells were incubated in the dark (Figures 6 A and 7 A, lanes 13 to 20, NER). Repair of CPDs was quantified as time-dependent increase of the fraction of intact restriction fragments (Figures 6 B and 7 B). The results revealed efficient repair of YRpFT35 by both pathways in wild type and all H3 mutants (Figure 6 B). Thus, the *hht2* mutations did not generally affect DNA accessibility and repair in the minichromosomes. None of the H3 mutants were defective in NER. Repair was also efficient in the subtelomeric region of chromosome VII containing *URA3* (Figure 7 B). In this locus, however, repair by photolyase in wild type cells was slightly reduced compared with repair of the H3 mutants (Figure 7B, 15 minutes NER+PR) and NER might be mildly enhanced in the *hht2-*T80A mutant. This result correlates with loss of *URA3* silencing and reflects an altered accessibility in compact silenced and open non-silenced chromatin as shown previously [19]. Taken together, there were only subtle differences in CPD repair between wild type and *hht2* mutants, which provides additional *in vivo* evidence that the mutants do not generally affect DNA accessibility in chromatin.

## Discussion

We have studied the histone H3 mutations *hht2*-T80A, *hht2*-K79E, *hht2*-L70S, and *hht2*-E73D that alter amino acid residues in an area on the nucleosome surface with distinct roles in silencing and the DNA-damage response pathways [25], [26], [39], [41]. We show that those mutants have little effect on chromatin structures and conclude that they rather act by recruitment of proteins involved in silencing and DNA-damage response than by direct or factor mediated nucleosome interactions and positioning.

Structural studies pointed out that histone residues (and their posttranslational modifications) on the solvent exposed nucleosome surface might impact higher order structures by promoting interactions between nucleosomes in chromatin arrays and within condensed chromatin structures [3], [4], [6], [58]. Here, we used a minichromosome system to investigate nucleosome positioning *in vivo*, since previous studies established that the nucleosome positions in those constructs are not determined by the DNA sequence, but mainly by NFRs acting as boundaries and chromatin folding [9], [10]. Moreover, those minichromosomes contained nucleosomes in close proximity, may be in face-to-face contact (tetranucleosomes), and, hence, were considered to be sensitive to mutations affecting the nucleosome surface. However, none of the H3 mutants showed effects on nucleosome positioning in the minichromosomes, nor on the supercoil density, the general sensitivity to MNase, or the accessibility to DNA repair enzymes. In addition, none of the H3 mutants tested here revealed an effect on nucleosome repeat length of genomic chromatin. Similarly, a *H3*-K79R mutant studied previously revealed very little differences in MNase accessibility compared to wildtype H3 [59]. Thus, at this level of analysis, there is no indication that the H3 residues studied so far significantly contribute to the general organization of chromatin. Furthermore, these results are consistent with the structural analysis of methylated H3-K79, which indicates that the effects of this modification are limited to localized structural changes to the nucleosome surface, and do not impact higher-order structures [60].

To test whether the impact of the mutants on silencing was related to changes in chromatin or recruitment of factors, we investigated the subtelomeric region containing *URA3* as a reporter gene. With the exception of the promoter nucleosome, the footprints of nucleosomes in the *URA3* gene were unchanged in the *hht*-mutants and *sir3*-deletion strains and slightly enhanced repair of the whole subtelomeric domain was observed in the mutants compared with the wild type. Those observations are entirely consistent with our previous studies using silencing deficient *sir3*-deletion strains that fail to recruit the silencing complexes [19]. We take this as an indication that the histone H3 residues act by binding factors rather than by changing chromatin structures at the level of nucleosomes. Our interpretation is consistent with genetic and biochemical evidence supporting direct interactions of the nucleosome core surface surrounding H3-K79 (the LRS surface) with the bromo-adjacent homology (BAH) domain of Sir3 to establish silencing [27], [31], [33], [34].

Several of the H3 mutants (L70S, T80A, E73D, Q76R) showed enhanced UV-sensitivity indicating a role in the DNA-damage response. Epistasis analysis of UV-survival with NER mutants (*rad1*) indicated that L70S and T80A act within the NER pathway, while E73D and Q76R revealed partially additive effects as shown for *dot1* [39], [41]. Our measurements of CPD repair by NER in presence and absence of photoreactivation, however, revealed no obvious difference between T80A, E73D and K79E, neither in minichromosomes nor in the subtelomeric region. Hence, neither the CPD accessibility (assayed by photoreactivation) nor the NER capacity appeared to be severely compromised. This observation does not support the hypothesis that histone H3 residues might define a binding site for repair factors [41] facilitating damage recognition and NER. However, deficiencies in NER have been reported for an *H3*-K79R mutation at silent mating locus *HML*, suggesting that the role of this domain in NER may be locus-specific and dependent on local chromatin structure [59]. Epistasis analysis of these mutations indicates that this domain additionally operates within other UV-damage response pathways [41], thus it is reasonable to anticipate that repair factors that bind to this domain in H3 may exist in these pathways. Consistent with this, DNA damage checkpoint factor Rad9 has been suggested to bind to methylated H3-K79 in response to double-stranded breaks [61] but additional studies are needed to determine if the interaction is direct or mediated by an additional nucleosome-binding recruitment factor, or if such an interaction arises in response to UV damage.

## Materials and Methods

### Yeast strains

The following previously created *S. cerevisiae* strains were utilized: JTY34U (YCpJT34 [*CEN4 ARS1 LYS2 HHF2-HHT2*]), JTY307U (YCpJTH3-7 [*CEN4 ARS1 LYS2 HHF2-hht2*-T80A]), JTY308U (YCpJTH3-8 [*CEN4 ARS1 LYS2 HHF2-hht2*-K79E]), JTY309U (YCpJT309 [*CEN4 ARS1 LYS2 HHF2-hht2*-L70S]), and JTY319U (YCpCF1 [*CEN4 ARS1 LYS2 HHF2-hht2*-E73D]) are all *MAT* **a** *ade2-101 (och) his3Δ200 lys2-801 (amb) trp1Δ901 ura3-52 adh4Δ::URA3-*TEL-VII-L *(hhf1-hht1)Δ::LEU2 (hhf2-hht2)Δ::HIS3* [26], [62]. MFY15, MFY16, MFY17, MFY18, and MFY19 were derived from JTY34U, JTY307U, JTY308U, JTY309U, and JTY319U, respectively, by transformation with the minichromosome YRpFT35 as described [10]. MFY57, MFY58, MFY59, MFY60, and MFY61, were derived from JTY34U, JTY307U, JTY308U, JTY309U, and JTY319U, respectively, by transformation with the minichromosome YRpFT38 as described [10]. KMY1 (*sir2Δ::TRP1*), KMY5 (*sir3Δ::TRP1*), and KMY9 (*sir4Δ::LEU2*) are all *MAT* **a** *ade2-101(och) his3Δ200 leu2Δ1 lys2-801 (amb) trp1Δ1 ura3-52 URA3-*TEL-V-R and were generated by deletion of *SIR2*, *SIR3*, or *SIR4*, respectively, using the plasmids GA604, pKL12, or GA391, respectively (kindly provided by S. Gasser). MFY62 (*MATα ade2-1 can1-100 his3-11,15 leu2-3,112 trp1Δ1 ura3-52 yku70Δ::HPH^r^*) was a gift from R. Wellinger. All transformations were verified by Southern blot hybridization.

### Chromatin analysis by micrococcal nuclease

Yeast cells were grown at 30°C in 5 liters synthetic complete (SC) dropout media lacking tryptophan to an *A_600_* of about 0.8–1.2 and either a crude nuclei extract was prepared essentially as described [63] and suspended in buffer A (20 mM Tris, pH 8.0; 150 mM NaCl; 5 mM KCl; 1 mM EDTA; 1 mM PMSF) (Figures 1 C and 3) or spheroplasts were prepared as described [64] and suspended in buffer B (20 mM Tris, pH 8.0; 150 mM NaCl; 5 mM KCl; 1 mM EDTA; 0.15% NP40; 1 mM PMSF; 1 µg/ml pepstatin; 1 µg/ml leupeptin) (Figures 1 A, 1 D, and 2). 2 ml aliquots of the crude nuclei extract or 400 µl aliquots of the spheroplasts were supplemented with CaCl_2_ to a final concentration of 1 mM and digested with micrococcal nuclease (1–200 U/ml, Roche Diagnostics) at 37°C for 5 minutes. In case of the crude nuclei extracts, the reactions were terminated by addition of 3 ml 2.5× buffer G2 (2 M guanidine HCl; 75 mM Tris, pH 8.0; 75 mM EDTA; 12.5% Tween-20; 1.25% Triton X-100; 200 mg/ml RNase A (Sigma); 300 mg/ml Proteinase K (Roche Diagnostics)) and incubation for 2 h at 50°C. Genomic DNA was isolated using Genomic-tips 100/G (QIAGEN), and dissolved in 10 mM Tris, pH 8.0; 1 mM EDTA. In the case of the digested spheroplasts, the reactions were terminated by addition of 40 µl stop solution (10% SDS; 100 mM EDTA) and the DNA was isolated and dissolved in 10 mM Tris, pH 8.0; 1 mM EDTA.

### Mapping micrococcal nuclease cutting sites by indirect end labeling

The DNA was cut with XbaI or HhaI to map the minichromosomes and *adh4Δ::URA3*-TEL-VII-L, respectively, electrophoresed on 1% agarose-TBE (89 mM Tris-borate; 2 mM EDTA; pH 8.3) gels, transferred to Zeta-Probe GT membranes, and hybridized with ^32^P-labeled DNA probes [65]. Probes were generated by random hexanucleotide-primed DNA synthesis using a HexaLabel DNA labeling Kit (Fermentas), [α-^32^P]CTP (Amersham Biosciences), and short DNA templates as indicated in the Figure legends. MNase digestion patterns were analyzed using a PhosphorImager screen (Amersham Biosciences) and ImageQuant software (Molecular Dynamics).

### Plasmid DNA topology analysis

Superhelical density of the minichromosomes YRpFT35 was determined as described [66]. Briefly, yeast cells were grown at 30°C in SC dropout media lacking tryptophan to an *A_600_* of about 1–1.5, mixed with an equal volume of buffered ethanol/toluene (20 mM Tris, pH 8.0; 95% ethanol; 3% toluene) prechilled to −20°C followed immediately by addition of 0.5 M EDTA to a final concentration of 10 mM. Spheroplasts were prepared by incubation with Zymolyase 100T (Seikagaku Kogyo Co., Ltd.) for 30 minutes at 30°C. The DNA was isolated using Genomic-tip 100/G (QIAGEN), electrophoresed on a 0.75% agarose-TBE gel containing 1 µg/ml chloroquine (Sigma) and transferred to Zeta-Probe GT membranes. YRpFT35 topoisomers were probed with a ^32^P-labeled EcoRI-XbaI fragment of the *TRP1* gene. Topoisomers were analyzed using a PhosphorImager.

### Telomere length analysis

Genomic DNA was prepared from yeast strains grown in YPD to an *A_600_* of about 2. DNA was digested with XhoI, separated on a 1% agarose-TBE gel, transferred to a Zeta-Probe GT membrane, and hybridized with a ^32^P-labeled DNA probe. The probe was generated by random hexanucleotide-primed DNA synthesis as described above using a short Y′ specific DNA template, which was generated by PCR from genomic yeast DNA using the primers Y′-1 (5′-TGCCGTGCAACAAACACTAAATCAA-3′) and Y′-3 (5′-CGCTCGAGAAAGTTGGAGTTTTTCA-3′).

### UV irradiation and repair by photolyase and nucleotide excision repair

UV irradiation of yeast cultures and repair of UV lesions was done as described previously [67]. Briefly, yeast cultures were grown in 6 liters SC dropout media lacking tryptophan at 30°C to a density of about 1×10^7^ cells/ml, resuspended in SD (0.67% yeast nitrogen base without amino acids, 2% dextrose) to about 3×10^7^ cells/ml. Suspensions were irradiated with UV light by use of Sylvania G15T8 germicidal lamps (predominantly 254 nm) at a dose of 150 J/m^2^ (measured by an UVX radiometer; UVP Inc., Upland, Calif.) and supplemented with adenine, uracil, and the appropriate amino acids. For photoreactivation and NER, the cell suspension was exposed to photoreactivating light (Sylvania type F15 T8/BLB bulbs, peak emission at 375 nm) at ∼1.3 mW/cm^2^ (measured by an UVX radiometer with a 365 nm photocell) and 27–30°C. For NER alone an aliquot was incubated in the dark at room temperature. Samples were chilled on ice, genomic DNA was isolated using Genomic-tips 100/G (QIAGEN), and dissolved in 10 mM Tris, pH 8.0; 1 mM EDTA. All steps until lysis of cells were done in yellow light (Sylviania GE Gold fluorescent light) to prevent undesired photoreactivation.

### CPD analysis by indirect end labeling

DNA was digested with XbaI or HhaI and nicked at CPDs with T4-endonuclease V (Epicentre) in 50 mM Tris, pH 7.5; 5 mM EDTA or mock treated with the same buffer. The DNA was electrophoresed on 1.5% alkaline agarose gels, transferred to Zeta-Probe GT membranes, and hybridized with ^32^P-labelled strand specific DNA probes. Strand-specific probes were generated by primer extension using the DNA fragments described in the Figure legends, [α-^32^P]CTP (Amersham Biosciences), and Taq DNA polymerase (Fermentas) for 20 cycles. The membranes were analyzed using a PhosphorImager and ImageQuant software. The CPD content (CPDs/top strand and CPDs/bottom strand) was calculated using the Poisson expression −ln(RFa/RFb), where RFa and RFb represent the signal intensity of the intact restriction fragment of the T4-endonuclease V and mock-treated DNA, respectively [68]. CPD repair was expressed as percentage with respect to the initial damage (0 min = 100% damage).
